# Structuring and De-Structuring of Nanovectors from Algal Lipids: Simulated Digestion, Preliminary Antioxidant Capacity and In Vitro Tests

**DOI:** 10.3390/pharmaceutics14091847

**Published:** 2022-09-01

**Authors:** Ilaria Clemente, Stefania Lamponi, Gabriella Tamasi, Liliana Rodolfi, Claudio Rossi, Sandra Ristori

**Affiliations:** 1 Department of Biotechnology, Chemistry and Pharmacy & Center for Colloids and Surface Science (CSGI), University of Siena, Via Aldo Moro 2, 53100 Siena, Italy; 2 Department of Agriculture, Food, Environment and Forestry (DAGRI), University of Florence, Piazzale delle Cascine 18, 50144 Florence, Italy; 3 Department of Chemistry “Ugo Schiff” & Center for Colloids and Surface Science (CSGI), University of Florence, Via della Lastruccia 3, 50019 Sesto Fiorentino, Italy

**Keywords:** lipid nanocarriers, algal biomass, curcumin, simulated digestion, antioxidant capacity, in vitro tests

## Abstract

Biocompatible nanocarriers can be obtained by lipid extraction from natural sources such as algal biomasses, which accumulate different lipid classes depending on the employed culture media. Lipid aggregates can be distinguished according to supramolecular architecture into lamellar and nonlamellar structures. This distinction is mainly influenced by the lipid class and molecular packing parameter, which determine the possible values of interfacial curvature and thus the supramolecular symmetries that can be obtained. The nanosystems prepared from bio-sources are able to self-assemble into different compartmentalized structures due to their complex composition. They also present the advantage of increased carrier-target biocompatibility and are suitable to encapsulate and vehiculate poorly water-soluble compounds, e.g., natural antioxidants. Their functional properties stem from the interplay of several parameters. Following previous work, here the functionality of two series of structurally distinct lipid nanocarriers, namely liposomes and cubosomes deriving from algal biomasses with different lipid composition, is characterized. In the view of their possible use as pharmaceutical or nutraceutical formulations, both types of nanovectors were loaded with three well-known antioxidants, i.e., curcumin, α-tocopherol and piperine, and their carrier efficacy was compared considering their different structures. Firstly, carrier stability in biorelevant conditions was assessed by simulating a gastrointestinal tract model. Then, by using an integrated chemical and pharmacological approach, the functionality in terms of encapsulation efficiency, cargo bioaccessibility and kinetics of antioxidant capacity by UV-Visible spectroscopy was evaluated. Subsequently, in vitro cytotoxicity and viability tests after administration to model cell lines were performed. As a consequence of this investigation, it is possible to conclude that nanovectors from algal lipids, i.e., cubosomes and liposomes, can be efficient delivery agents for lipophilic antioxidants, being able to preserve and enhance their activity toward different targets while promoting sustained release.

## 1. Introduction

One of the main drawbacks in the assessment of the biological and pharmacological activity of natural drugs is their low solubility in physiological media, which results in poor bioavailability. Many attempts have been made to improve the absorption of these molecules by living organisms to improve their efficacy [1,2,3,4,5]. An effective strategy involves encapsulation in soft matter nanocarriers [6], particularly lipid-based vectors.

Lipids are the class of biological molecules with richest polymorphism that results in a wide range of supramolecular aggregates. This characteristic has been exploited to obtain customized nanosystems in soft matter science and has been applied mainly, but not exclusively, to drug delivery, food science and the cosmetic/consumer goods industry [7,8,9]. Lipid nanosystems can be classified on the basis of their supramolecular architecture in two large categories, i.e., lamellar and nonlamellar assemblies [10] Among these systems, lamellar aggregates are characterized by the presence of one or multiple bilayers self-assembled with vesicular morphology. Vesicles and liposomes are to date the most commonly employed nanocarriers in research and pharmaceutical applications, especially in dilute conditions, due to their easiness of design, modeling and similarity to existing biological membranes [11,12,13]. Among nonlamellar structures, cubic aggregates, constituted of highly curved lipid surfaces draped around water channels, are also very popular. In diluted regime, cubosomes differ from liposomes for their pronounced inner ordering and more complex structure. This makes their design and characterization more laborious, though many advantages can stem from their use, such as higher surface-to-volume ratio, enhanced cargo encapsulation and sustained delivery [14,15,16]. As discussed in a previous work [16], a rich variety of biocompatible nanosystems can be obtained from natural biomasses to be applied as drug vectors with improved sustainability and compatibility for bioactive compounds [17,18]. Particularly, biomasses from microalgae are advantageous for such purposes [19,20,21] thanks to the possibility of obtaining different lipid compositions by modifying algal metabolism [22] towards the production of higher percentages of either triglyceride or phospholipid content [23,24,25]. These lipid classes self-assemble preferentially into cubic aggregates or into lamellar vesicles, respectively [10,26,27]. While biomasses from natural sources allow high (up to 70–80%) lipid yields, [25,28] possible drawbacks may be found in the characterization of complex lipid mixtures. However, these nanovectors represent ideal carriers for natural drugs such as antioxidant molecules able to exert protective action against targeted diseases induced by oxidative stress pathways, e.g., cancer, atherosclerosis, Systemic Inflammatory Response Syndrome (SIRS), neurodegenerative diseases. Particularly interesting among such compounds, curcumin (1,7-bis(4-hydroxy-3methoxyphenyl)-1,6-heptadien-3,5-dione) is a polyphenol usually derived from the rhizome of *Curcuma longa* L. These plants have been used in Asian countries for centuries in traditional medicine as curative herbs for numerous pathologies due to their anti-radical, anti-inflammatory, anti-mutagenic, anti-microbial and anti-cancer activities [29,30]. α-Tocopherol is another popular antioxidant and, together with γ-tocopherol, is the most abundant vitamin E component that can be taken from the diet. In vivo, dietary requirements of vitamin E are currently limited to α-tocopherol because this is the only form that reverses vitamin E deficiency [31]. Moreover, α-tocopherol is known to inhibit key events in inflammatory signaling [32]. Finally, the natural antioxidant piperine is a simple alkaloid isolated from the seeds of *Piper nigrum*. Piperine and its derivatives exhibit a wide range of pharmacological and biological properties, such as anti-tumor, antioxidant, anti-inflammatory, anti-mycobacterial, insecticidal activities, etc. [33].

Here, curcumin, α-tocopherol and piperine were used as model nutraceuticals and encapsulated in nanovectors to obtain fully biocompatible formulations. The nanoformulations were prepared using lipids extracted from the marine microalga *Nannochloropsis* sp. cultivated in two different conditions, i.e., with or without nitrogenated compounds in the culture medium [16,24]. This led to different lipid composition in the starting material, which generated two distinct structural arrangements of the obtained nanoaggregates, one series exhibiting cubic symmetry and the other a more standard unilamellar structure [34]. The bioactive compounds (curcumin, α-tocopherol and piperine) were loaded either singularly or combining curcumin with one of the other two molecules as adjuvant, to investigate the possible synergistic properties arising from their interaction in a confined environment [16]. Liposome and cubosome nanocarriers showed different behavior not only on the basis of their structure but also depending on the loaded bioactives. Therefore, this work was focused on the investigation of the concurring action of structure and synergism of loaded compounds, to identify the factors influencing drug delivery capability and efficiency. The carrier stability and cargo bioaccessibility were assessed by simulated digestion tests through a gastrointestinal tract model (GIT) with similarities to the physiological environments of stomach and intestine, e.g., pH, temperature and bile salts with enzymes addition. The antioxidant capability and bioactivity of the formulations were tested preliminarily towards different targets, i.e., the well-known ABTS radical and model NIH3T3 fibroblast lines. 

## 2. Materials and Methods

### 2.1. Materials

Curcumin (C), piperine (P) and α-tocopherol (T) and all the organic solvents were purchased from Sigma-Aldrich (St. Louis, MO, USA) and used as received. *Nannochloropsis oceanica* F&M-M24 was obtained from the Fotosintetica & Microbiologica (F&M) S.r.l. culture collection and characterized as previously reported (see Supplementary Materials) [16,25,35]. Dulbecco’s Modified Eagle’s Medium (DMEM), trypsin solution and all the solvents used for cell culture were purchased from Lonza (Verviers, Belgium). Mouse immortalized fibroblasts NIH3T3 were from the American Type Culture Collection. The biorelevant medium containing bile salts and surfactants was purchased from Biorelevant.com Ltd., London, UK. Pepsin from porcine gastric mucosa (600 U/mg) and pancreatin with 4× USP specification were purchased from Sigma-Aldrich.

### 2.2. Preparation of Cubosome and Liposome Nanovectors

The lyophilized and powdered algal biomasses, from nitrogen-sufficient and nitrogen-deprived culture media, respectively, were stored in freezer at −20 ± 1 °C and de-frozen before use, then lipid extraction was performed in Folch solution (CHCl_3_/CH_3_OH 2:1 *v*/*v*) by weighing 25 mg/mL of each biomass type, as previously described [16]. The solution was stirred for 24 h at room temperature, then the solvents were evaporated to obtain a lipid film. Stock solutions of the three antioxidants in acetone were added to the dry film. Subsequent evaporation was performed under vacuum to obtain a dry lipid film, which was then rehydrated, using MilliQ water and equilibrated for 12 h. The lipid water suspensions were then subjected to extensive vortexing followed by eight freeze-and-thaw cycles. Finally, high-power sonication using a Bandelin Sonopuls HD 4050 with 20 kHz (Bandelin electronic GmbH & Co., Berlin, Germany) was employed to downsize the lipid nanovectors, as previously described [16]. The antioxidant molecules not associated with nanovectors were part of a small precipitate or stuck to the walls of the glassware. In either case, they were discarded by filtering the samples through polycarbonate membranes with 0.5 mm pore size.

The complete series of systems studied in this work is described in Table 1.

### 2.3. Encapsulation Efficiency

As reported in a previous work [16], the encapsulation efficiency (EE%) of the loaded compounds was quantified by UV–Vis spectroscopy. The samples were properly diluted in ethanol to disrupt the nanovectors and release the entrapped molecules [16]. Absorption spectra were recorded at room temperature on a Thermo Scientific Evolution 220 UV–Vis spectrophotometer, equipped with a xenon flash lamp and double-beam geometry, using 10 mm path length quartz cuvettes. Absorption maxima at 425 nm, 350 nm and 270 nm were chosen for curcumin, piperine and α-tocopherol, respectively. The quantification was carried out recording the absorbances of standard solutions, in dynamic range of linearity (curcumin 0.92–3.68 mg/L; α-tocopherol, 2.15–8.6 mg/L; piperine, 0.285–2.85 mg/L) and calibration curves showing correlation factors R^2^ > 0.990 were accepted for subsequent analyses. All spectra were recorded in triplicate and averaged (n = 3). The EE% of nanovectors was calculated as follows:

(1) $$Encapsulation\;efficiency\;\left(\%\right)=\frac{loaded\;amount}{tot\;amount}\times 100$$

### 2.4. Stability and Release in Digestion Conditions

Stability and release experiments on nanovectors subjected to digestion conditions in a gastrointestinal (GIT) model [36,37,38] simulating the gastric and intestinal environments were carried out in a commercial biorelevant Simulated Gastric Fluid/Simulated Intestinal Fluid (SGF/SIF) medium (Biorelevant.com Ltd.), with the addition of digestion enzymes, i.e., pepsin and pancreatin respectively. The protocol reported by Chen et al. [37] was employed with few modifications. Briefly, the samples were diluted 1:10 *v*/*v* and heated 5–10 min at 37 degrees under magnetic agitation, then a 1:1 *v*/*v* pre-heated volume of SGF with pepsin 3.2 mg/mL was added. The pH was adjusted to 2.5 and the mixture was incubated for 1 h at 37 degrees with magnetic agitation. After one hour the digestion was stopped bringing the pH at 7, then an aliquot was taken to analyze curcumin release at time t = 1 h. The remaining volume was mixed with a 1:1 *v*/*v* SIF medium containing 2 mg/mL pancreatin and the pH was adjusted to 7. Then the mixture was kept for 2 h at 37 degrees under magnetic agitation, and sampling of digesta were taken at 30, 60, 90 and 120 min [37,38]. The digestion was stopped by rapid freezing in dry ice. All samplings were carried out in duplicate. 

UV-Vis spectroscopy measures allowed evaluation of both the release of curcumin in the digesta samples at various time intervals and its bioaccessibility at the end of the digestion process through the GIT model [39]. Digesta aliquots at different times were centrifuged at 5000 rpm for 30 min, then the clear supernatant, which constitutes the micellar phase where the digested curcumin is solubilized, was taken for analysis [37,39]. Curcumin was extracted by adding EtOH (1:500 final dilution) to obtain absorption intensity at 425 nm in an appropriate range. The cumulative release was then calculated from a known calibration curve using Equation (2) and normalizing samples on the basis of their previously measured EE%.

(2) $$Cumulative\;release\;\left(\%\right)=\frac{Abs\;sample}{Abs\;Std}\times 100$$

The bioaccessibility % of curcumin after the whole digestion process (time t = 180 min in Figure S2) was calculated according to the Equation (3):

(3) $$Bioaccessibility\;\left(\%\right)=\frac{C_{Micell}}{C_{Raw\;digesta}}\times 100$$

where $C_{Micell}$ is the curcumin concentration in the micellar phase and $C_{Raw\;digesta}$ is the curcumin retrieved from nanovectors at the end of the digestion process in the raw digesta [37,38,39]. 

### 2.5. ABTS Assay: Kinetic of Absorbance Decrement of ABTS^•+^ Radical Treated with Nanovectors in Two Different Conditions

The ABTS assay was employed to evaluate the kinetics of antioxidant capacity of the lipid nanocarriers both in EtOH (that disrupts the lipid structures) and simply diluted in water, for at least 7 h. The protocol previously described by Bonechi et al. [40] was used with some modifications. Briefly, ABTS^•+^ free radical cation was prepared by treating a solution of ABTS (7 mM) with a K_2_S_2_O_8_ solution (140 mM) and incubating overnight (12–16 h in the darkness at 4 ± 1 °C). Then it was properly diluted in absolute EtOH or MilliQ water before use. A known volume was then treated with Trolox standard solutions (ranging 0–20.00 μM) for calibration. After 30 min of incubation, the adsorption at 734 nm was recorded with a UV–Vis spectrometer against EtOH as reference. The nanovector samples were initially diluted 1:100 (in EtOH for kinetics of disrupted nanovectors, in water for kinetics of unbroken nanovectors) to avoid interference from algae material absorbance (matrix effect). Then 20 μL of each sample were added to 1.00 mL of ABTS^•+^ and 80 μL of EtOH or water (final sample dilution 1:5500). Calibration curves were built reporting the relative decreasing in absorbance (Abs_734_%) of the ABTS^•+^ solution treated with standards or samples, with respect to the blank solution, according to Equation (4):

(4) $${\mathrm{Abs}}_{734}\;\left(\%\right)=\left[1-\left(\frac{Abs_{Std/Spl}}{Abs_{Blk}}\right)\right]\times 100$$

Calibration curves were plotted as ∆Abs% vs. Trolox standard concentration and the ones showing correlation factors R^2^ > 0.990 were accepted for analysis. The Trolox samples stability was checked during time. All measurements were recorded in triplicate and averaged (n = 3).

### 2.6. In Vitro Cytotoxicity Evaluation

The in vitro cytotoxicity of the test samples was determined by the direct contact test following the standard ISO 10995-5:200942 by using fibroblasts NIH3T3. Cells were cultured in DMEM supplemented with 10% (*v*/*v*) fetal calf serum, 4 mM L-glutamine, 100 IU/mL penicillin, 100 µg/mL streptomycin and 1% (*v*/*v*) MEM non-essential amino acid solution at 37 °C in a humidified atmosphere containing 5% CO_2_. Once at 80% confluence, and after washing with PBS 0.1 M, cells were detached by trypsin-EDTA solution (0.5% trypsin in 0.53 mM EDTA) and then centrifuged at room temperature for 5 min at 1500 rpm. The pellet was resuspended in complete medium in order to have a cell density of 1.5 × 10^3^/mL of complete DMEM.

Then, one mL of cell suspensions was seeded in each well of 24 multiwell plate and incubated at 37 °C in 5% CO_2_ for 24 h. At the end of the incubation, the DMEM was discharged from each well and the test compounds diluted in complete medium were added. The in vitro cytotoxicity of both empty nanovectors and nanovectors loaded with antioxidant molecules (C, P and T) at different concentrations was evaluated by Neutral Red Uptake (NRU) assay after 24 h of incubation following the procedure already reported [41]. The tested nanocarriers concentrations were 0.1, 0.01 and 5.0% *v*/*v*, containing different curcumin, piperine and α-tocopherol concentrations depending on encapsulation efficiency. The in vitro cytotoxicity of increasing concentrations of C- and P- and T-free solution in ethanol was also evaluated in order to assess how the encapsulation process was able to influence cell viability.

### 2.7. Antioxidant Activity Evaluation

Fibroblasts NIH3T3 were pre-incubated with various hydrogen peroxide concentrations (1, 5, 10, 25, 30, 50, 75, 100 μM) for 15 min at 37 °C in 5% CO_2_. Then, the H_2_O_2_ was removed, cells were washed with PBS 0.1 M, rinsed with DMEM containing various ratios of the tested substances and incubated at 37 °C in 5% CO_2_ for 24 h. Then, cell viability was determined by NRU assay [41].

### 2.8. Statistical Analysis

Cell assays were repeated three times in six replicates. Results of cell viability experiments were reported as mean ± standard deviation (SD) and analyzed by one-way ANOVA. Fisher’s test was performed in order to assess individual differences (*p* < 0.05).

## 3. Results and Discussion

The in-depth characterization of drug delivery systems for pharmacological applications involves both the study of the physico-chemical properties, i.e., morphology and interactions at molecular level, and the investigation of functional properties resulting from these attributes. Considering the relevance of efficacy assessment for pharmacological and nutraceutical applications, here the attention was focused on the analysis and comparison of the formulations’ capability as delivery vectors in biomimetic environments.

### 3.1. Simulated Gastrointestinal Digestion and Curcumin Release

**Figure 1:**
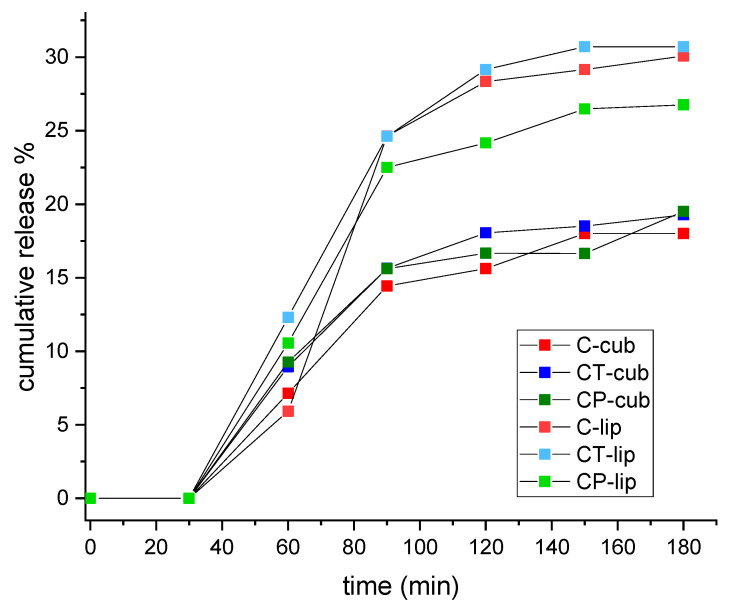
Cumulative release % curves throughout 3 h total SGF/SIF digestion time.

The stability of the nanovectors was tested in a lipid-specific and biorelevant environment, i.e., a simulated digestive medium. Figure 1 shows the curves of cumulative curcumin release from the nanosystems subjected to digestion, as a result of the occurring destabilization. Generally, samples of the liposome series showed higher percentages of cumulative release and bioaccessibility at the end of the digestion process, while cubosome samples showed a slower and sustained release resulting from the attack of digestive enzymes. This could be due to different lipid composition (triglycerides vs. phospholipids) and/or to the lipid accessibility to enzyme attack which depended on the supramolecular arrangement. In both liposomal and cubic systems, release percentages were rather low after 60 min (Figure 1), which corresponds to the passage through the gastric tract. This indicated low levels of digestion and curcumin bioavailability in the stomach and high stability of the two aggregate types. On the other hand, a marked increment of released curcumin was observed during the first 30 min in the intestinal medium (total t = 90 min), followed by less significant increments, ultimately leading to a plateau in the last samplings. Such results confirmed that both liposomes and cubosomes were highly resistant to gastric pH conditions and enzymes, as pepsin could only metabolize the small protein content from the algal biomass, and they were able to protect curcumin in such an environment whereas most of the digestion happened in the intestinal tract due to the pancreatic enzymes. Nevertheless, even though the first 30 min in the SIF marked high digestive activity, all samples preserved some stability and structure, keeping up cargo release until the end of the process (t = 180 min) and showing a final percentage around 30%. Figure S1 compares the two series of aggregates showing the percentage of curcumin bioaccessibility at the end of the process. Liposomal nanovectors generally showed higher percentages, likely due to easiness of attack from enzymes and lower ability in rearranging their structure upon disassembling.

### 3.2. Kinetics of Decrement of Absorbance of ABTS^•+^ Treated with Both Regular and Disrupted Nanovectors

**Figure 2:**
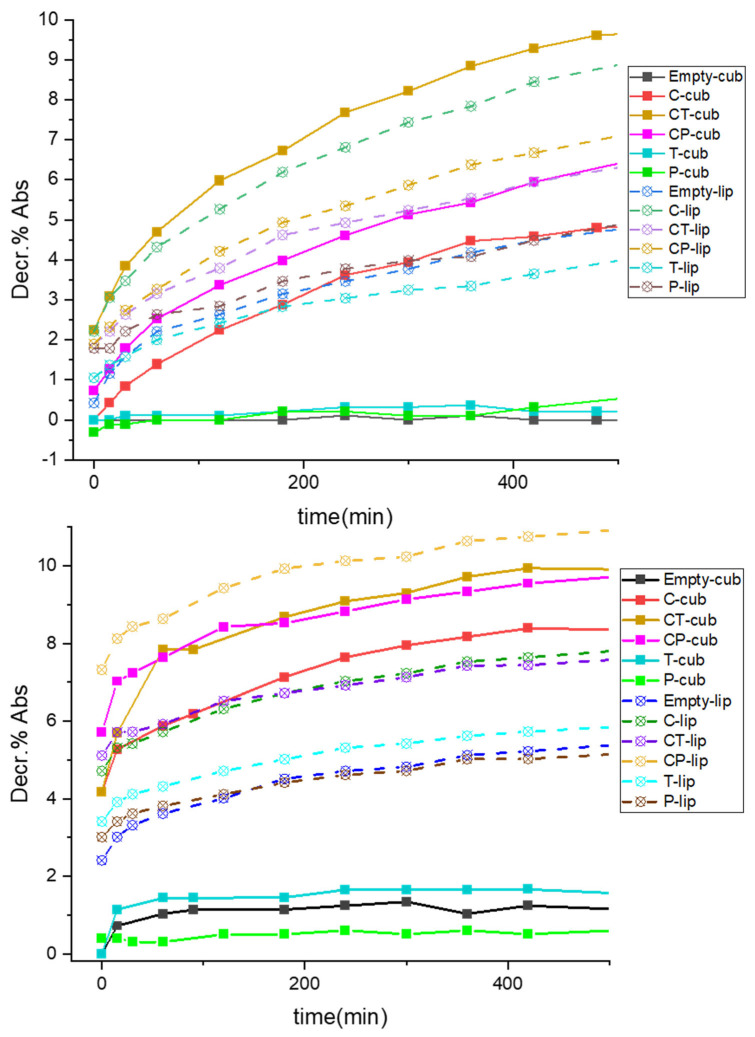
Kinetic of decrement of absorbance for sample in standard (i.e., unbroken) conditions, solid squares for cubosomes, empty circles for liposomes, (**up**); and in disrupted conditions, solid squares for cubosomes, empty circles for liposomes (**down**).

Two different approaches were employed to study the antioxidant power of these formulations, i.e., testing both regular nanovectors in their standard, unbroken condition, and in a disrupted state triggered by ethanol. In this latter case, the loaded molecules were released in the solvent due to carrier rupture. All samples were added to the ABTS^•+^ radical cation solution, as described in Materials and Methods section, and their antioxidant capacity was evaluated with respect to Trolox as a standard. For unbroken nanovectors, the kinetic of insertion of the radical in the bilayer, a necessary step to reach the cargo, was investigated together with the spatial localization and accessibility of the molecules inserted in the lipid environment. In fact, for the first measurements until 30 min, all the samples showed almost no decrement of absorbance (decr. %), except for CT-cub and CP-cub for the cubosome series and the three curcumin samples (C-lip, CT-lip and CP-lip) for the liposome series (Table 1, Figure 2 and Tables S3 and S4). All these samples showed steeper curves with higher decrement values. In the former case, a synergistic/cooperative effect between the two antioxidants and curcumin could be evinced; indeed, CT-cub and CP-cub showed the highest values of decr. % (Figure 2, Table S3). In cubosome systems, this could be also related to the higher EE% of the two samples with adjuvants with respect to C-cub sample (Tables S2 and S3). Moreover, for this series it appeared that the synergy between curcumin and α-tocopherol provoked a greater effect than piperine, since even though CP-cub showed higher EE%, the curve for CT-cub revealed a higher decr. % (Figure 2, Table S3). For these nanovectors in regular conditions, this could be due to the different spatial localization and mobility of the loaded molecules in the bilayer, making them less or more accessible to the radical. Concerning the liposome series, on the contrary, the sample with only curcumin (C-lip) always showed greater decr. % with respect to samples with either of the two adjuvants combined with curcumin (Figure 2, Table S3). Apparently, in these aggregates curcumin had lower synergy with α-tocopherol and piperine, likely due to less accessibility of the molecules enveloped in the bilayer, oppositely to the cubic structure. Moreover, Empty-lip showed some antioxidant capacity, in contrast to Empty-cub that did not show significant effect of decr. %, possibly due to compositional differences in the employed biomasses. While both series of samples containing curcumin showed comparable values of decr. %, in the case of nanocarriers containing only α-tocopherol or piperine quite low values were revealed if compared to samples containing curcumin alone or with adjuvants.

The same protocol was applied to study the kinetic of disrupted nanovectors in ethanol. In both sample series, cubosomes and liposomes, a progressive increase of decr. % Abs value was noticeable, starting right away from t = 0 (Figure 2), predictably more pronounced for samples with curcumin and curcumin combined with one of the two adjuvants. As already observed in standard conditions for cubosomes, despite the higher EE% of CP-cub, the two samples had superposed values of decr. % (Figure 2) showing a stronger antioxidant effect for CT-cub. The liposomes also displayed cooperative effects for both CT-lip and CP-lip, which despite having smaller EE% of C-lip (Table S2), showed equal or higher decr. % values, differently from what was seen above. It is likely that the molecules, not being entrapped in the lamellar bilayer as in standard conditions, could interact with each other more efficiently. As already seen above, also in disrupted conditions in the liposome nanocarriers the cooperative effect seemed larger for the curcumin-piperine combination (CP-lip in Figure 2, Table S4), while the samples containing only α-tocopherol or piperine (T-lip and P-lip) showed more pronounced decr. % with respect to their cubosome counterparts.

Some considerations can be made on the two nanovectors series from the data obtained on antioxidant capacity in either regular or disrupted conditions, plotted against the EE% of each sample (bar plot in Figure S2, only most relevant samples shown). Since the radical–antioxidant interaction modality was dependent either on radical insertion or on effective interactions during release, the antioxidant capacity was mainly influenced by the carrier structure, the guest molecules, or both, according to the test conditions. Concerning the cubosome series, CT-cub showed more antioxidant power in both conditions (Figure S2). This could depend on enhanced synergy of curcumin with tocopherol than with piperine when inserted in the cubic bilayer, or it could be due to the observed higher encapsulation of tocopherol when combined with curcumin, or finally to different availability when the guest molecules are encapsulated. The contrary was true for sample CP-lip in disrupted state (Figure S2), likely due to more effective interactions in the liposomal bilayer, whereas in regular conditions liposomes performed better without adjuvants. This effect was attributed to accessibility issues, as discussed above.

### 3.3. Evaluation of NIH3T3 Cytotoxicity

The evaluation of cytotoxicity was performed by the quantitative determination of neutral red incorporation by fibroblasts NIH3T3 after 24 h of exposure to various doses of C, P, and T.

**Figure 3:**
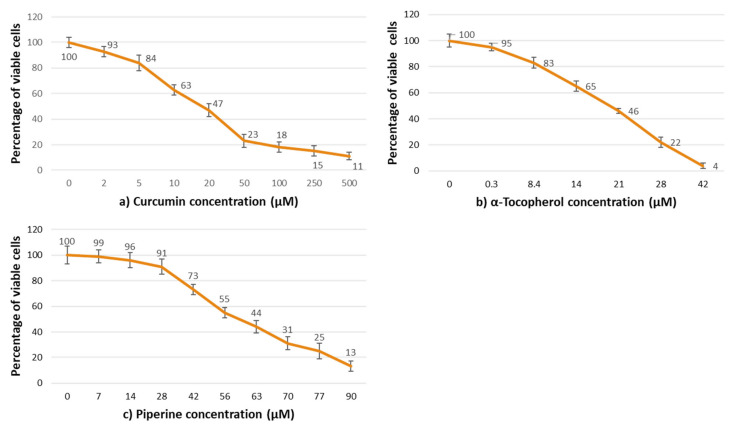
Percentage of viable fibroblasts NIH3T3 after 24 h of contact with different concentrations of: (**a**) Curcumin; (**b**) α-Tocopherol; (**c**) Piperine.

Figure 3 shows the effect of increasing concentrations of standard solutions of C, T and P with respect to the 100% viability of control (complete DMEM). For concentrations more than 20 M, the viability of the cell in contact with C (Figure 3a) was significantly reduced, with a 50% loss in percentage of viable cells. The identical result was seen for T and P, respectively, at 21 µM (Figure 3b) and 56 µM (Figure 3c).

The cytotoxicity of the two nanovectors systems, liposomes and cubosomes, at three different concentration values (0.1, 1.0 and 5.0%) was also evaluated. The results are reported in Figure S3. The cubosome nanovectors lacked any cytotoxic effect towards NIH3T3. On the contrary, the liposomes appeared to be able to significantly reduce cell viability by increasing their concentration. This toxic effect of liposome nanocarriers as a function of their concentration can be probably ascribed to a mechanic effect [42]. In fact, at the highest tested concentration, i.e., 1.0 and 5.0% (*v*/*v*), the nanovectors completely covered the cell’s surface (see Figure S4), likely due to enhanced adhesion and lamellar stacking effect. Cubosome nanocarriers loaded with C, T, P, CT and CP were chosen for cell cultures experiments since liposomes showed cytotoxic effects at two of the tested concentrations (Figure S3). The Empty-cub sample showed no cytotoxic effect at all three concentrations tested, nor did the presence of the antioxidant molecules confer any cytotoxic effect.

### 3.4. Antioxidant Activity Analysis and Discussion

Hydrogen peroxide solution with concentrations ranging from 1 to 100 µM was tested on NIH3T3 fibroblasts to determine the concentration–effect relationship. In particular, the decreasing of cell viability after treatment with H_2_O_2_ followed by contact with different concentrations of free C, T and P solutions, and empty and loaded cubosome nanocarriers, was determined in order to assess the ability of cubosomes to reverse the toxic effect of H_2_O_2_. Liposomes were not tested, since they showed cytotoxic effect at two of the tested concentrations, i.e., 1 and 5% (*v*/*v*).

**Figure 4:**
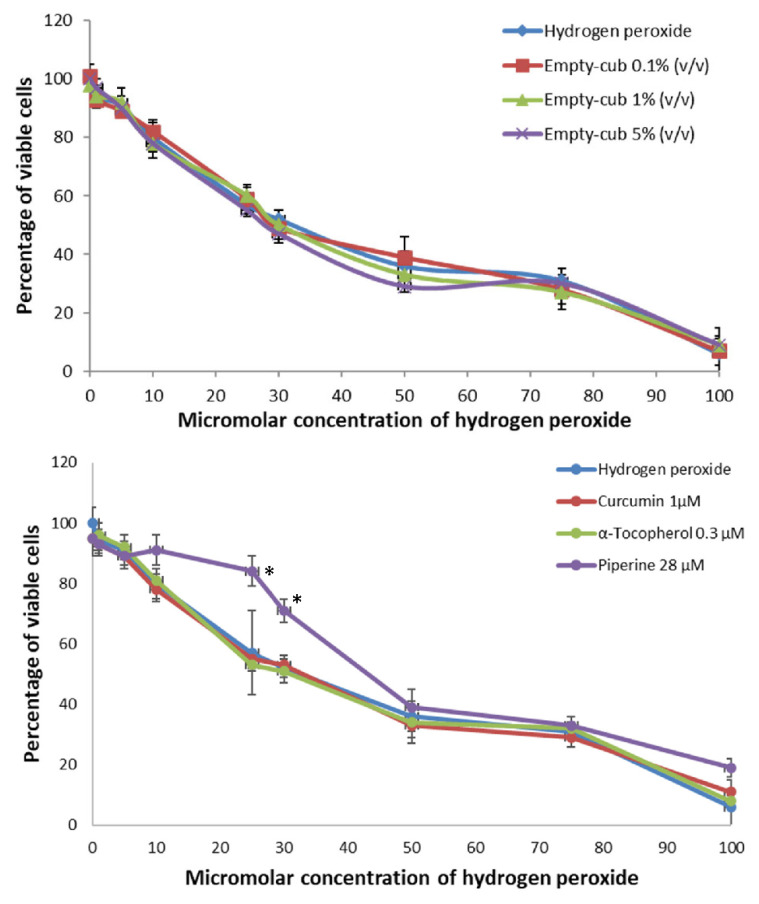
Top to bottom, Antioxidant activity of Empty-cub nanocarriers. The nanocarriers do not have any effect towards hydrogen peroxide at the tested concentrations. No values were statistically different in comparison to hydrogen peroxide. Antioxidant activity of non-cytotoxic concentrations of solutions of Curcumin, α-Tocopherol and Piperine. * Values are statistically different versus hydrogen peroxide *p* < 0.05.

Figure 4 shows that cell viability decreased by increasing hydrogen peroxide concentration in presence of Empty-cub nanovectors. The same trend was observed for solutions of free curcumin, 1 µM, and α-tocopherol, 0.3 µM (Figure 4). On the contrary, piperine, 28 µM, increased the percentage of viable cells at 5, 10, 25 and 30 µM H_2_O_2_ concentrations, but not at higher values, showing the protection of cells from H_2_O_2_ pre-treatment and reducing cell death.

**Figure 5:**
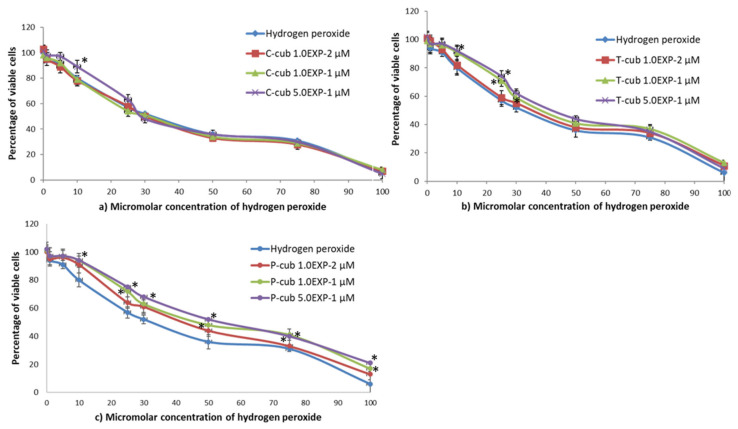
Antioxidant activity of (**a**) C-cub, (**b**) T-cub, (**c**) P-cub, see Table S2 in Supplementary Materials for concentration and EE% values. * Values are statistically different versus hydrogen peroxide *p* < 0.05.

The C-cub nanovectors (EE% in Table S2) at the highest curcumin concentration tested (5.0 × 10^−1^ µM) showed antioxidant activity towards hydrogen peroxide concentration of 1, 5, 10 and 25 µM (*p* < 0.01; ANOVA) (Figure 5). The presence of α-tocopherol (EE% in Table S2) gave antioxidant activity to cubosomes at the molecule concentration of 1.1 × 10^−1^ and 5.2 × 10^−1^ µM towards hydrogen peroxide concentration of 1, 5, 10, 25 and 30 µM (*p* < 0.01) (Figure 5). Finally, cubosomes loaded with piperine (EE% in Table S2) at all the concentration values tested showed antioxidant activity towards hydrogen peroxide concentration of 1, 5, 10, 25, 30 and 50 µM (*p* < 0.01).

**Figure 6:**
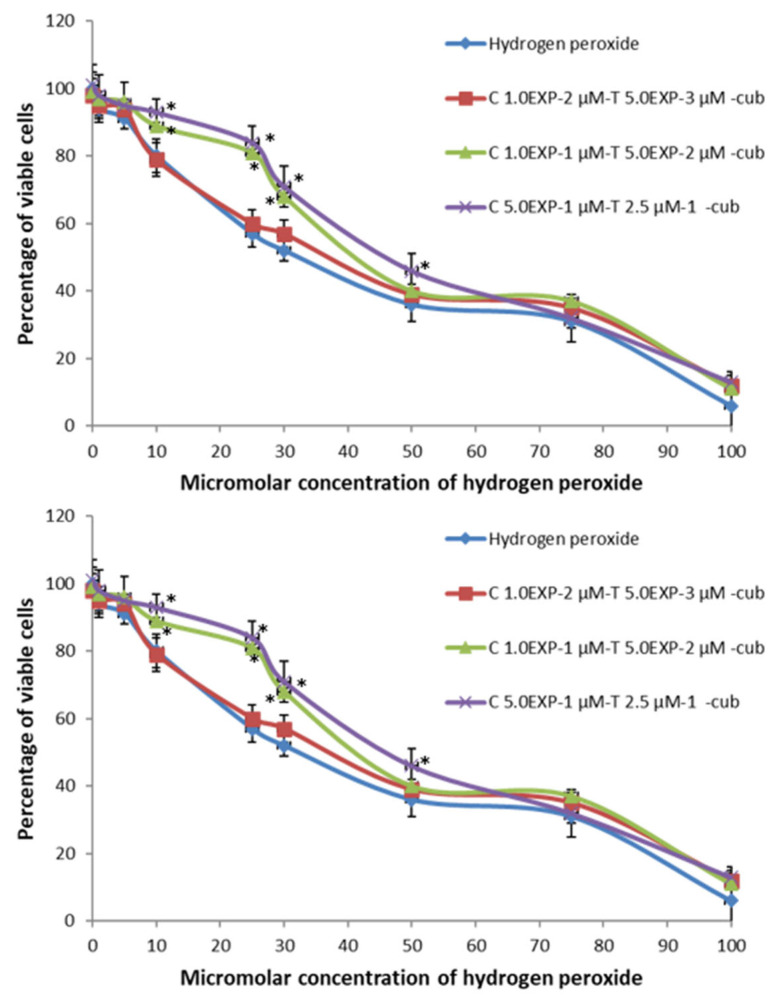
Top to bottom, antioxidant activity of CT-cub, CP-cub, see Table S2 in Supplementary Materials for concentration and EE% values. * Values are statistically different versus hydrogen peroxide *p* < 0.05.

The loading of cubosomes with both curcumin/α-tocopherol (CT-cub) or curcumin/piperine (CP-cub) increased the ability of the nanosystems to protect cells from hydrogen peroxide pre-treatment as shown in Figure 6. In both nanosystems, the lowest concentration tested of the molecules (i.e., curcumin 1.0 × 10^−2^ µM/α-tocopherol 5.0 × 10^−3^ µM and curcumin 1.0 × 10^−2^ µM/piperine 5.0 × 10^−3^ µM) lacked any protective effect (Figure 6). On the contrary, both curcumin 1.0 × 10^−1^ µM/α-tocopherol 5.0 × 10^−2^ µM and curcumin 1.0 × 10^−1^ µM/piperine 5.0 × 10^−2^ µM protected cells from the toxic effect of hydrogen peroxide pre-treatment at concentrations of 10, 25 and 30 µM. Finally, the highest antioxidant molecules concentrations tested, i.e., curcumin 5.0^−1^ µM/α-tocopherol 2.5 × 10^−1^ µM and curcumin 5.0 × 10^−1^ µM/piperine 2.5 × 10^−1^ µM, exerted a protective effect also at H_2_O_2_ concentration value of 50 µM. These results showed that the loading of nanocarriers with two molecules improved their antioxidant activity towards cell systems thanks to a synergic effect, confirming the results obtained from the ABTS assay.

## 4. Conclusions

In this work, two series of lipid nanocarriers with slightly different lipid composition, which resulted in structurally distinct liposome and cubosome assemblies, were used to deliver commercially popular nutraceuticals, i.e., three well-known antioxidants and their combinations. In particular, their functionality in biorelevant environments was evaluated. The study of the functional properties of different formulations was carried out following both a chemical and pharmacological approach, which confirmed the complex nature of these nanocarriers stemming from various contributions, particularly their supramolecular structure and cargo molecules. Simulated digestion tests were carried out to assess the stability of cubosomes and liposomes and the bioaccessibility of curcumin at the end of the process. These experiments evidenced the ability of both nanocarrier types to protect antioxidants from degradation until absorption in the intestinal tract. Here the carrier functionality was mostly influenced by the aggregate composition and architecture, due to different susceptibility to enzyme attack. On the other hand, the combination of aggregate type and guest molecules influenced the antioxidant capability of the formulations. Regarding the kinetics of ABTS^•+^ radical cation quenching, it was shown that all systems were able to induce a decrement of absorbance either in regular or disrupted conditions, with different interaction ability especially in the case of curcumin co-loaded with piperine or α-tocopherol. Comparing the two series, cubosome samples showed generally higher quenching in disrupted conditions, probably due to higher encapsulation efficiency, whereas in regular conditions liposomes performed better due to easiness of radical insertion (possibly resulting in higher accessibility). The structural arrangement of the carrier was also shown to be relevant for the cytotoxicity of these formulations. Specifically, cubosomes gave excellent performance displaying total absence of toxicity at any tested concentration. On the contrary, liposomes showed cytotoxicity towards fibroblasts except at the lowest concentration, due to a mechanical effect of adhesion and lamellar stacking on the cell surface which led to poor cell viability. Hydrogen peroxide-induced oxidative stress tests on fibroblasts showed that α-tocopherol, and, even more, piperine, encapsulated alone in cubosomes acted as good antioxidants on H_2_O_2_-treated cells. This was less evident toward the ABTS^•+^ radical, likely due to localization and accessibility issues. This result allowed formulation of the hypothesis of lower CP-cub efficacy with respect to CT-cub when the carriers were not disrupted (despite higher EE%) due to lower accessibility of piperine to the radical. On the contrary, CP-cub performed slightly better in H_2_O_2_ cell tests, showing that the administration conditions concur to functionality. Hydrogen peroxide tests fully supported the evidence of synergic effects of curcumin with either of the two adjuvants in the confined bilayer space, boosting its antioxidant capacity towards both radicals and cells. In more quantitative terms, these trials showed that the concentration of biomolecules in the nanocarriers needed to increase cell viability is at least one order of magnitude lower than the concentration needed for the free antioxidant molecules. In conclusion, the present study evidenced that algae-derived lipid nanocarriers can be effective systems to deliver poorly water-soluble antioxidant molecules, both improving sustained administration and enhancing synergistic properties. It also showed that functional properties ultimately result from the interplay of various parameters, stemming from both structure and interactions at molecular level.

## Figures and Tables

**Table 1:** Sample names, structure [34] and loaded compound for the systems used in this study. Samples loaded with only tocopherol or piperine were employed for antioxidant capacity assay and cell tests for comparison, in order to evaluate the antioxidant effect of these molecules alone and then their synergic action with curcumin.

Sample Name	Supramolecular Structure	Loaded Compound	Loaded Concentration
Empty-cub	cubosome	-	-
C-cub	cubosome	Curcumin (C)	10^−2^ M
CT-cub	cubosome	C+ α-tocopherol (T)	C at 10^−2^ M, T at 5 × 10^−3^ M
CP-cub	cubosome	C+ piperine (P)	C at 10^−2^ M, P at 5 × 10^−3^ M
T-cub	cubosome	T	5 × 10^−3^ M
P-cub	cubosome	P	5 × 10^−3^ M
Empty-lip	liposome	-	-
C-lip	liposome	C	10^−2^ M
CT-lip	liposome	C+T	C at 10^−2^ M, T at 5 × 10^−3^ M
CP-lip	liposome	C+P	C at 10^−2^ M, P at 5 × 10^−3^ M
T-lip	liposome	T	5 × 10^−3^ M
P-lip	liposome	P	5 × 10^−3^ M

## Supplementary Materials

The following supporting information can be downloaded at: https://www.mdpi.com/article/10.3390/pharmaceutics14091847/s1. Table S1. Fatty acid composition (%) of the three lipid classes, i.e. neutral lipids (NL), glycolipids (GL), and phospholipids (PL) of starved Nannochloropsis oceanica F&M-M24 biomass; Table S2. Dynamic Light Scattering measurements for tested samples; Table S3. Encapsulation efficiency (EE%) of curcumin (C), tocopherol (T) and piperine (P) expressed as mean ± standard deviation (SD) of measurements in triplicate (n = 3), relative standard deviation (RSD%) and drug loading (DL%), determined by UV–Vis for all the samples employed in these experiments. Software OriginPro 2021 was used for analysis. It can be noticed that when samples were loaded only with one of the two adjuvants to be tested for comparison purposes, they mostly showed lower values of EE% than those seen for curcumin alone, likely indicating lower host-guest compatibility; Table S4. Decr. % Abs values, standard deviation and RSD for all tested samples in standard condition. The values are the average of triplicate measures; Table S5. Decr. % Abs values, standard deviation and RSD for all tested samples in disrupted condition. The values are the average of triplicate measures; Figure S1. SAXS measurements on tested samples showed the presence of unilamellar liposomes (left) from the biomass containing phospholipids and cubosomes (right) from the one containing triglycerides; Figure S2. Curcumin bioaccessibility % values of all tested samples after 3 h total SGF/SIF digestion time. Higher bioaccessibility can be seen for the liposome samples with respect to cubosome ones, likely due to easier access to enzyme attack; Figure S3. Bar plot showing the Decr. % Abs ± standard deviation of relevant samples in either regular or disrupted state against EE%; Figure S4. Fibroblasts viability after 24 h of contact with different concentrations of E-lip and cubosome nanovectors both empty and loaded with the different test molecules. * Data are statistically different in comparison to negative control (complete culture medium) *p* < 0.01; Figure S5. Optical microscope images of NIH3T3 mouse fibroblasts after 24 h of contact with: (a) negative control (complete medium); (b) 0.1% *v*/*v* cubosome nanocarriers; (c) 0.1% *v*/*v* liposome nanocarriers; (d) after removing the liposome nanocarrier.

## References

[1] Esposito E., Sguizzato M., Drechsler M., Mariani P., Carducci F., Nastruzzi C., Valacchi G., Cortesi R. (2019). Lipid nanostructures for antioxidant delivery: A comparative preformulation study. Beilstein J. Nanotechnol..

[2] Vaz S., Silva R., Amaral M.H., Martins E., Sousa Lobo J.M., Silva A.C. (2019). Evaluation of the biocompatibility and skin hydration potential of vitamin E-loaded lipid nanosystems formulations: In vitro and human in vivo studies. Colloids Surf. B Biointerfaces.

[3] Araya-Sibaja A.M., Wilhelm K., González-Aguilar G.A., Vega-Baudrit J.R., Salazar-López N.J., Domínguez-Avila J.A., Navarro-Hoyos M. (2021). Curcumin Loaded and Co-loaded Nanosystems: A Review from a Biological Activity Enhancement Perspective. Pharm. Nanotechnol..

[4] Ternullo S., Gagnat E., Julin K., Johannessen M., Basnet P., Vanic Z., Skalko-Basnet N. (2019). Liposomes augment biological benefits of curcumin for multitargeted skin therapy. Eur. J. Pharm. Biopharm..

[5] Xu Y., Liu H., Song L. (2020). Novel drug delivery systems targeting oxidative stress in chronic obstructive pulmonary disease: A review. J. Nanobiotechnol..

[6] Milcovich G., Antunes F., Golob S., Farra R., Grassi M., Voinovich D., Grassi G., Asaro F. (2016). Thermo-responsive hydrogels from cellulose-based polyelectrolytes and catanionic vesicles for biomedical application. J. Biomed. Mater. Res. Part A.

[7] Hafez I.M., Cullis P.R. (2001). Roles of lipid polymorphism in intracellular delivery. Adv. Drug Deliv. Rev..

[8] Luzzati V., Tardieu A., Gulik-Krzywicki T. (1968). Polymorphism of Lipids. Nature.

[9] Müller R.H., Radtke M., Wissinga S.A. (2002). Nanostructured lipid matrices for improved microencapsulation of drugs. Int. J. Pharm..

[10] Larsson K., Fontell K., Krogh N. (1980). Structural relationships between lamellar, cubic and hexagonal phases in monoglyceride–water systems. Possibility of cubic structures in biological systems. Chem. Phys. Lipids.

[11] Gregoriadis G. (2008). Liposome research in drug delivery: The early days. J. Drug Target..

[12] Allen T.M. (2000). Solving Drug Delivery Problems with Liposomal Carriers. Controlled Drug Delivery.

[13] Sebaaly C., Greige-Gerges H.C. (2019). Lipid Membrane Models for Biomembrane Properties’ Investigation. Current Trends and Future Developments on (Bio-) Membranes.

[14] Abourehab M.A.S., Ansari M.J., Singh A., Hassan A., Abdelgawad M.A., Shrivastav P., Abualsoud B.M., Amaral L.S., Pramanik S. (2022). Cubosomes as an emerging platform for drug delivery: A review of the state of the art. J. Mater. Chem. B.

[15] Barriga H.M.G., Holme M.N., Stevens M.M. (2019). Cubosomes: The next generation of smart lipid nanoparticles?. Angew. Chem. Int. Ed..

[16] Clemente I., Bonechi C., Rodolfi L., Bacia-Verloop M., Rossi C., Ristori S. (2021). Lipids from algal biomass provide new (nonlamellar) nanovectors with high carrier potentiality for natural antioxidants. Eur. J. Pharm. Biopharm..

[17] Corma A., Iborra S., Velty A. (2007). Chemical routes for the transformation of biomass into chemicals. Chem. Rev..

[18] Biermann U., Bornscheuer U., Meier M.A.R., Metzger J.O., Schäfer H.J. (2011). Oils and fats as renewable raw materials in chemistry. Angew. Chem. Int..

[19] Nacka F., Cansell M., Méléard P., Combe N. (2001). Incorporation of α-tocopherol in marine lipid-based liposomes: In vitro and in vivo studies. Lipids.

[20] Colzi I., Troyan A.N., Perito B., Casalone E., Romoli R., Pieraccini R., Škalko-Basnet N., Adessi A., Rossi F., Gonnelli C. (2015). Antibiotic delivery by liposomes from prokaryotic microorganisms: Similia *cum similis* works better. Eur. J. Pharm. Biopharm..

[21] Savaghebi D., Barzegar M., Mozafari M.R. (2019). Manufacturing of nanoliposomal extract from *Sargassum boveanum* algae and investigating its release behavior and antioxidant activity. Food Sci. Nutr..

[22] Annaliese F.K., Wong D.M., Danielewicz M.A., Anderson L.A., Boothe J.R. (2013). Phenotypic Screening with Oleaginous Microalgae Reveals Modulators of Lipid Productivity. ACS Chem. Biol..

[23] Spoehr H.A., Milner H.W. (1949). The chemical composition of Chlorella: Effect of environmental conditions. Plant Physiol..

[24] Suen Y., Hubbard J.S., Holzer G., Tornabene T.G. (1987). Total lipid production of the green alga Nannochloropsis sp. QII under different nitrogen regimes. J. Phycol..

[25] Bondioli P., Della Bella L., Rivolta G., Chini Zittelli G., Bassi N., Rodolfi L., Casini D., Prussi M., Chiaramonti D., Tredici M.R. (2012). Oil production by the marine microalgae Nannochloropsis sp. F&M-M24 and Tetraselmis suecica F&M-M33. Bioresour. Technol..

[26] Israelachvili J.N., Mitchell D.J., Ninham B.W. (1976). Theory of self-assembly of hydro-carbon amphiphiles into micelles and bilayers. J. Chem. Soc. Faraday Trans. 2 Mol. Chem. Phys..

[27] Kulkarni C.V. (2012). Lipid crystallization: From self-assembly to hierarchical and biological ordering. Nanoscale.

[28] Hu Q., Sommerfeld M., Jarvis E., Ghirardi M., Posewitz M., Seibert M., Darzins A. (2008). Microalgal triacylglycerols as feedstocks for biofuel production: Perspectives and advances. Plant J..

[29] Pulido-Moran M.M., Jorge Moreno F., Ramirez-Tortosa C., Ramirez-Tortosa M.C. (2016). Curcumin and health. Molecules.

[30] Duan D., Doak A.K., Nedyalkova L., Shoichet B.K. (2015). Colloidal Aggregation and the in Vitro Activity of Traditional Chinese Medicines. ACS Chem. Biol..

[31] Bruno R.S., Mah E., Vitamin E. (2014). Reference Module in Biomedical Sciences.

[32] Brigelius-Flohé R., Traber M.G. (1999). Vitamin E: Function and metabolism. FASEB J..

[33] Qu H., Lv M., Xu H. (2015). Piperine: Bioactivities and structural modifications. Mini Rev. Med. Chem..

[34] Clemente I. (2022). Compartmentalized Algal-Based Nanocarriers as Vectors for Antioxidants: Structural and Functional Characterization. Ph.D. Thesis.

[35] Menicucci F., Michelozzi M., Raio A., Tredici M., Cencetti G., Clemente I., Ristori S. (2021). Thymol-loaded lipid nanovectors from the marine microalga Nannochloropsis sp. as potential antibacterial agents. Biocatal. Agric. Biotechnol..

[36] McClements D.J., Li Y. (2010). Review of in vitro digestion models for rapid screening of emulsion-based systems. Food Funct..

[37] Chen S., Li Q., McClements D.J., Han Y., Dai L., Mao L., Gao Y. (2020). Co-delivery of curcumin and piperine in zein-carrageenan core-shell nanoparticles: Formation, structure, stability and in vitro gastrointestinal digestion. Food Hydrocoll..

[38] Zou L., Zheng B., Zhang R., Zhang Z., Liu W., Liu C., Xiao H., McClements D.J. (2016). Food-grade nanoparticles for encapsulation, protection and delivery of curcumin: Comparison of lipid, protein, and phospholipid nanoparticles under simulated gastrointestinal conditions. RSC Adv..

[39] Cuomo F., Cofelice M., Venditti F., Ceglie A., Miguel M., Lindman B., Lopez F. (2018). In-vitro digestion of curcumin loaded chitosan-coated liposomes. Colloids Surf. B Biointerfaces.

[40] Bonechi C., Donati A., Tamasi G., Pardini A., Rostom H., Leone G., Rossi C. (2019). Chemical characterization of liposomes containing nutraceutical compounds: Tyrosol, hydroxytyrosol and oleuropein. Biophys. Chem..

[41] Lamponi S. (2022). Preliminary In Vitro Cytotoxicity, Mutagenicity and Antitumoral Activity Evaluation of Graphene Flake and Aqueous Graphene Paste. Life.

[42] Inglut C.T., Sorrin A.J., Kuruppu T., Vig S., Cicalo J., Ahmad H., Huang H.C. (2020). Immunological and Toxicological Considerations for the Design of Liposomes. Nanomaterials.

