# Supplementary material for: Structuring and De-Structuring of Nanovectors from Algal Lipids: Simulated Digestion, Preliminary Antioxidant Capacity and In Vitro Tests
Source: Pharmaceutics. 2022 Sep 1;14(9):1847. doi: 10.3390/pharmaceutics14091847 (PMC9500752; doi:10.3390/pharmaceutics14091847)
Supplement: Supplementary file 1 [file pharmaceutics-14-01847-s001.zip › pharmaceutics-1848267-supplementary.pdf]

Supporting Information for

Structuring and de-structuring of nanovectors from algal lipids: simulated digestion, preliminary antioxidant capacity and in vitro tests.

Ilaria Clemente[1], Stefania Lamponi[1], Gabriella Tamasi[1], Liliana Rodolfi[2], Claudio Rossi[1], Sandra Ristori[3]

[1]Department of Biotechnology, Chemistry and Pharmacy & CSGI, University of Siena, Via Aldo Moro 2, 53100 Siena, Italy
[2]Department of Agriculture, Food, Environment and Forestry (DAGRI), University of Florence, Piazzale delle Cascine, 18, 50144 Florence, Italy
[3]Department of Chemistry "Ugo Schiff" & CSGI, University of Florence, Via della Lastruccia 3, 50019 Sesto Fiorentino, Italy

1) Lipid composition of algal biomass

The results for the nitrogen starved biomass, as expected from the literature on *Nannochloropsis* and from previous analyses of our collaborators,[14,24,34] showed a strong prevalence of neutral lipids that constituted about 76% of the total, followed by glycolipids (19.37%) and phospholipids (4.73%) (Table S1). Considering TAGs are known for self-assembling in nonlamellar structures, these results gave insights on the plausible supramolecular architecture of the obtained nanosystems.

Regarding the fatty acids composition, the C 16:0 and C 16:1 n 7 chains that are typical in TAGs were the most representative in the neutral lipid fraction, together with C 18:1 n 9 (Table S1). The saturated palmitic acid was predominant in all the fractions, up to 62% of the phospholipid fraction, as seen in the literature.[34] Oleic acid (C 18:1 n 9; 26%), myristic acid (C 14:0; 17%) and stearic acid (C 18:0; 12%) were the most represented in the glycolipid fraction together with the palmitic acid. In the phospholipid fraction instead myristic acid (13%) was more abundant than oleic acid (10%). Long chain fatty acids (8.58%) were detected in lower amounts in the three fractions. Glycolipid and phospholipid fractions contained mostly saturated fatty acids, while on the contrary unsaturated fatty acids were more present in the neutral lipid fraction (Table S1).

Table S1. Fatty acid composition (%) of the three lipid classes, i. e. neutral lipids (NL), glycolipids (GL), and phospholipids (PL) of starved *Nannochloropsis oceanica* F&M-M24 biomass.

Fatty acid	Lipid class NL fraction 75.90%	GL fraction 19.37%	PL fraction 4.73%	TOTAL (%)
C 10:0	0.10	0.17	0.00	0.10
C 14:0	14.31	17.25	13.03	14.54
C 15:0	0.78	0.91	0.62	0.78
C 16:0	25.34	35.85	61.62	28.61
C 16:1 n 7	17.85	3.72	4.03	14.31
C 17:0	1.90	1.67	0.82	1.77
C 18:0	5.26	12.08	8.42	6.56
C 18:1 n 9 c	23.61	26.40	10.28	15.46
C 20:0	0.36	0.31	0.19	0.34
C 20:3 n 6	0.99	0.31	0.42	0.82
C 20:4 n 6	2.62	0.43	0.25	2.06
C 20:5 n 3	6.82	0.85	0.28	5.30
C 22:0	0.07	0.05	0.06	0.06
Total (%)	75.90	19.37	4.73	100.00
total saturated	48.10	68.29	84.75	
total unsaturated	51.90	31.71	15.25	

2) DLS measurements on tested samples

Table S2. Dynamic Light Scattering measurements for tested samples.

**Sample name**	**Mean size (nm) Peak 1**±SD	**Mean size (nm) Peak 2**±SD	**PdI**
Empty-cub	48±3	404±35	0.3
C1-cub	153±15	663±68	0.45
CT-cub	69±8	494±43	0.3
CP-cub	59±2	376±78	0.35
Empty-lip	133±20	2168±68	0.4
C1-lip	318±32	548±50	0.43
CT -lip	-	495±51	0.27
CP -lip	148±37	554±220	0.5

3) SAXS measurements

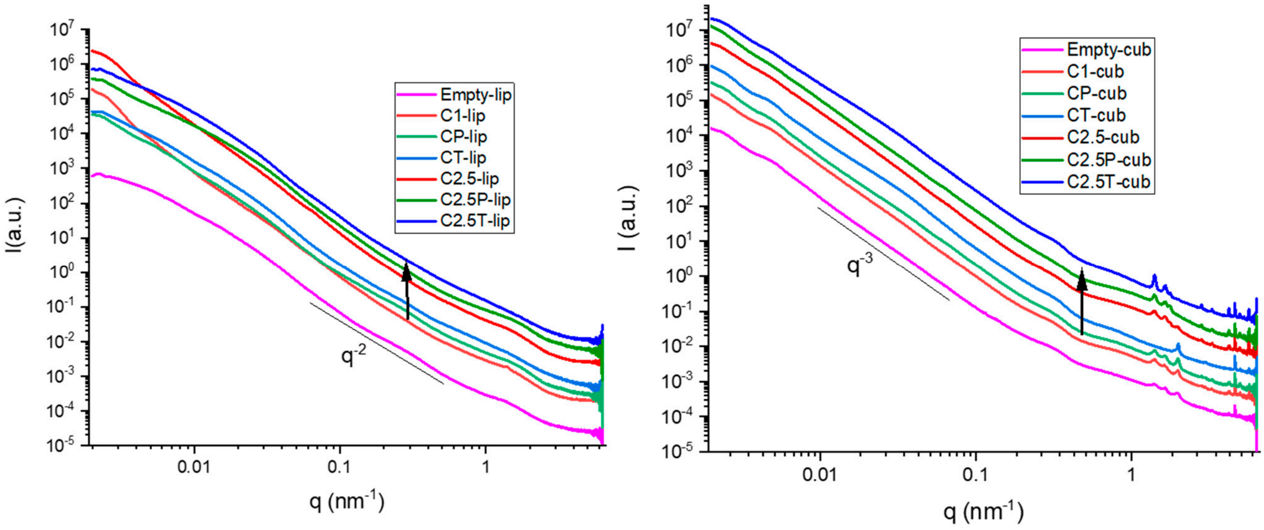

Figure S1. SAXS measurements on tested samples showed the presence of unilamellar liposomes (left) from the biomass containing phospholipids and cubosomes (right) from the one containing triglycerides.

4) Plot of curcumin bioaccessibility values after simulated digestion

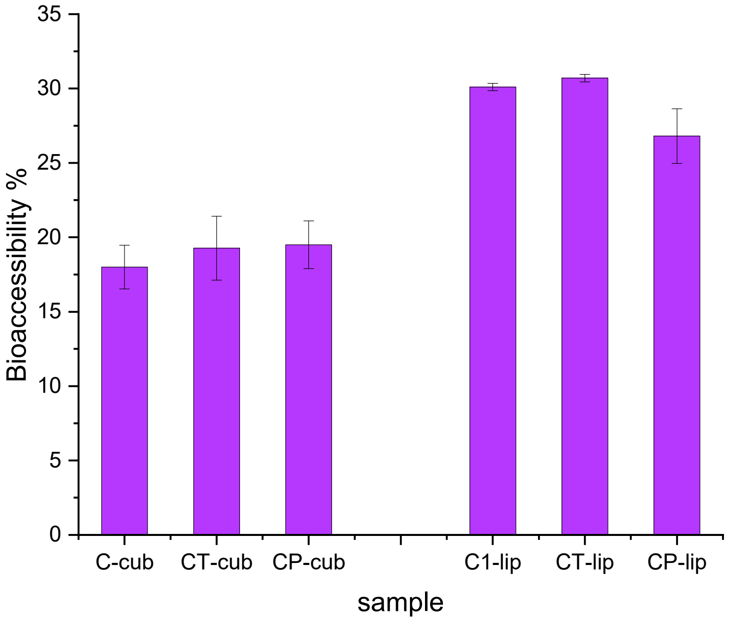

Figure S2. Curcumin bioaccessibility % values of all tested samples after 3 hours total SGF/SIF digestion time. Higher bioaccessibility can be seen for the liposome samples with respect to cubosome ones, likely due to easier access to enzyme attack.

5) Encapsulation efficiency of tested samples

Table S3. Encapsulation efficiency (EE%) of curcumin (C), tocopherol (T) and piperine (P) expressed as mean ± standard deviation (SD) of measurements in triplicate (n=3), relative standard deviation (RSD%) and drug loading (DL%), determined by UV–Vis for all the samples employed in these experiments. Software OriginPro 2021 was used for analysis. It can be noticed that when samples were loaded only with one of the two adjuvants to be tested for comparison purposes, they mostly showed lower values of EE% than those seen for curcumin alone, likely indicating lower host-guest compatibility.

Sample name	Loaded concentration	Encapsulation efficiency %±SD	RSD %	DL % cargo/ lipid ratio (mol/mol)
C-cub	10^{-2}M	59±6.06	11.39	19.7
CT-cub	C at 10^{-2}M; T at $5x10^{-3}$M	(C) 70.1±2.5<br>(T) 94.8±15.17	(C) 3.5<br>(T) 15.43	(C) 23.4<br>(T) 15.7
CP-cub	C at 10^{-2}M; P at $5x10^{-3}$M	(C) 80.7±1.08<br>P 78±4	(C) 1.34<br>(P) 5.27	(C) 26.9<br>(P) 13
T-cub	$5x10^{-3}$M	52.3±5	7.3	8.6
P-cub	$5x10^{-3}$M	26.8±0.36	1.35	4.5
C-lip	10^{-2}M	76.2±2.68	3.5	25.4
CT-lip	C at 10^{-2}M; T at $5x10^{-3}$M	(C) 65.7±4.3<br>(T) 75±6	(C) 6.58<br>(T) 8.2	(C) 22<br>(T) 12.5
CP-lip	C at 10^{-2}M; P at $5x10^{-3}$M	(C) 66.7±2.3<br>(P) 90±3	(C) 3.47<br>(P) 5.3	(C) 22.3<br>(P) 15
T-lip	$5x10^{-3}$M	54±4.2	7.8	9
P-lip	$5x10^{-3}$M	88±4.3	4.9	14.7

6) UV-Vis tables of decrement of absorbance values

Table S4. Decr. % Abs values, standard deviation and RSD for all tested samples in standard condition. The values are the average of triplicate measures.

Sample	Final Decr.% Abs	Stdev (decr.%)	RSD%
Empty-cub	-	-	-
C-cub	4.58	0.4	8.82
CT-cub	9.28	0.12	1.33
CP-cub	5.94	1.33	22.35
T-cub	0.21	0.06	29.31
P-cub	0.31	0.06	19.25
Empty-lip	4.48	0.27	6.15
C-lip	8.45	0.27	3.27

CT -lip	5.94	0.21	3.51
CP-lip	6.67	0.1	1.56
T-lip	3.65	0.73	20
P-lip	4.48	0.12	2.68

Table S5. Decr. % Abs values, standard deviation and RSD for all tested samples in disrupted condition. The values are the average of triplicate measures.

Sample	Final Decr.% Abs	Stdev (Decr.%)	RSD%
Empty-cub	1.24	0.18	14.46
C-cub	8.39	0.37	4.45
CT-cub	9.94	0.33	3.35
CP-cub	9.56	0.76	7.97
T-cub	1.66	0.06	3.6
P-cub	0.5	0.12	23.09
Empty-lip	5.2	0.31	5.87
C-lip	7.64	1.12	14.65
CT -lip	7.44	0.1	1.35
CP-lip	10.75	0.15	1.43
T-lip	5.73	0.15	2.68
P-lip	5.03	0.5	10.07

7) Bar plot of decrement of absorbance values

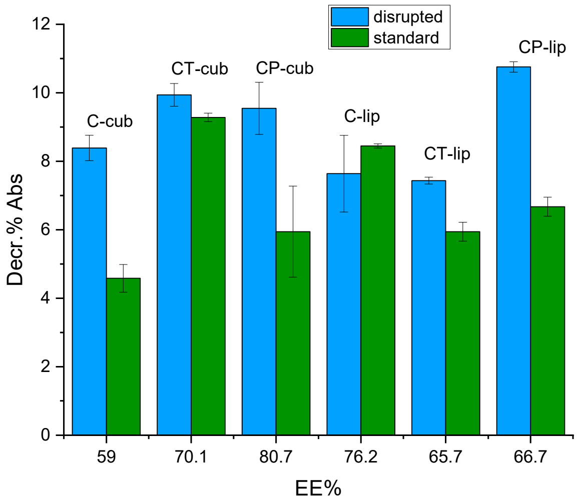

Figure S3. Bar plot showing the Decr. % Abs ± standard deviation of relevant samples in either regular or disrupted state against EE%.

8) Cytotoxicity test

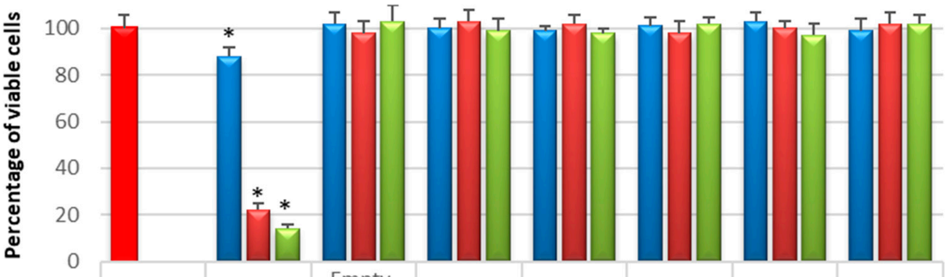

	Control	Empty-lip	Empty-cub	C-cub	CT-cub	CP-cub	T-cub	P-cub
0.10%	100	88	102	100	99	101	103	99
1%		22	98	103	102	98	100	102
5%		14	103	99	98	102	97	102

Samples

0.10% 1% 5%

Figure S4. Fibroblasts viability after 24 h of contact with different concentrations of E-lip and cubosome nanovectors both empty and loaded with the different test molecules. * Data are statistically different in comparison to negative control (complete culture medium) $p < 0.01$.

9) Optical microscope images showing fibroblasts viability.

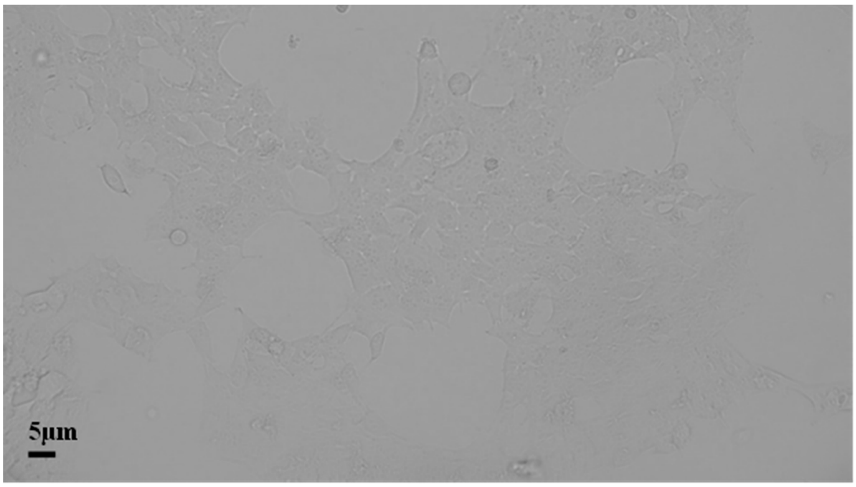

a)

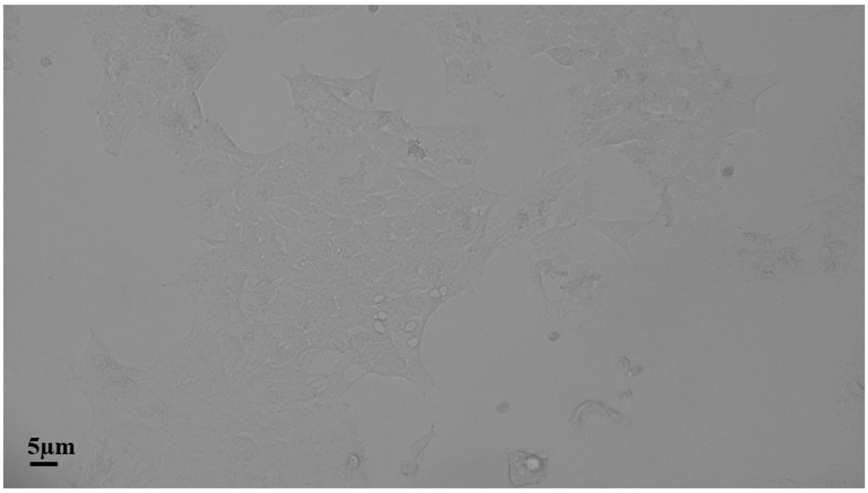

b)

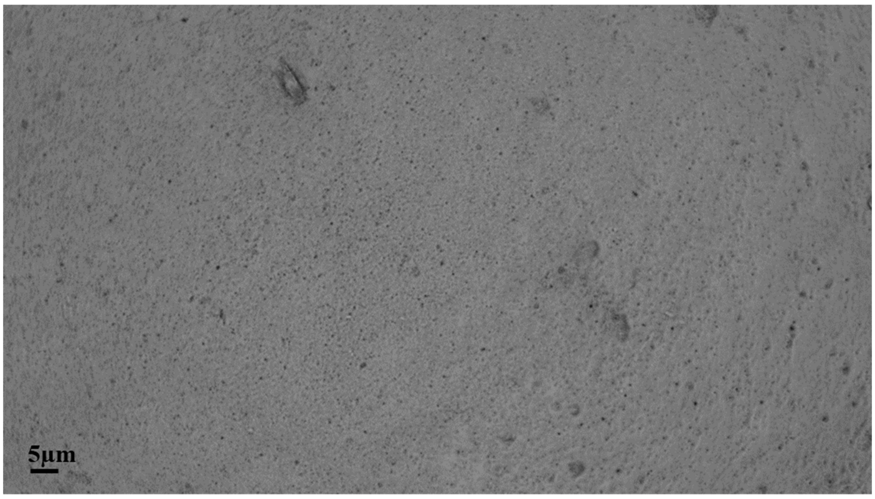

c)

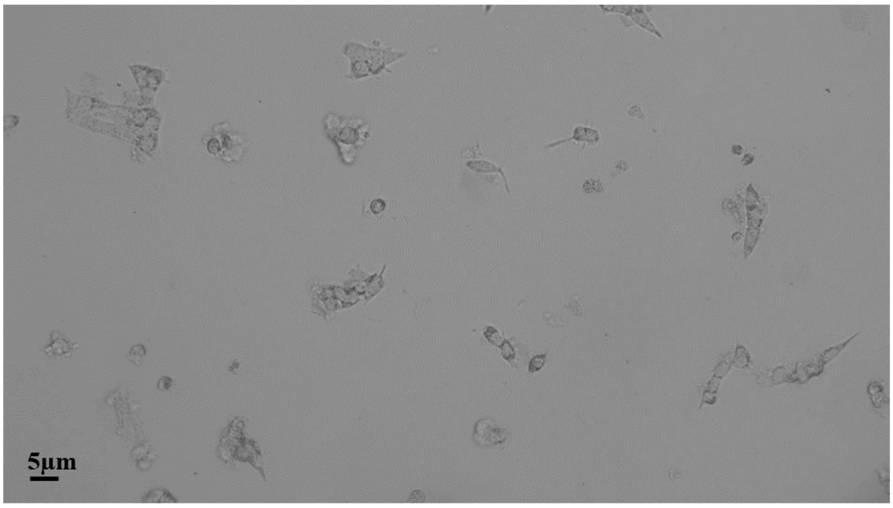

d)

Figure S5. Optical microscope images of NIH3T3 mouse fibroblasts after 24 hours of contact with: a) negative control (complete medium); b) 0.1% v/v cubosome nanocarriers; c) 0.1% v/v liposome nanocarriers; d) after removing the liposome nanocarrier.

Cells in contact with complete medium (Figure S5a) as well as those in presence of cubosome nanocarriers (Figure S5b) showed the normal fibroblastic morphology. When liposomal nanocarriers are added to the culture, the cells resulted completely covered by the lipidic vesicles (Figure S5c), due to enhanced adhesion and lamellar stacking effect on the cell surface upon administration of liposome samples. After removing the nanocarriers (Figure S5d) the cells resulted decreased and with altered morphology.
